# Genome-Wide Characterization of the *CAMTA* Gene Family in *Paulownia fortunei* and Potential Role of *PfCAMTA5* in Salt-Stress Responses

**DOI:** 10.3390/ijms27219476

**Published:** 2026-10-23

**Authors:** Yabing Cao, He Sun, Chunyang Liu, Jie Liu, Shenquan Cao, Zhenli Zhao

**Affiliations:** 1 Institute of Paulownia, Henan Agricultural University, Zhengzhou 450046, China; caoyb@henau.edu.cn (Y.C.); ehnus_023@163.com (H.S.); chyliu2013@163.com (C.L.); lj26292025@126.com (J.L.); 2 College of Forestry, Henan Agricultural University, Zhengzhou 450046, China; 3 College of Life Sciences, Henan Agricultural University, Zhengzhou 450046, China

**Keywords:** *Paulownia fortunei*, *CAMTA* gene family, salt stress, gene function

## Abstract

Calmodulin-binding transcription activator (CAMTA) transcription factors play key roles in plant stress responses, but their functions in *Paulownia fortunei* remain largely unknown. Here, we identified eight *CAMTA* family genes in *P. fortunei* and investigated their structural features, evolutionary relationships, and expression patterns under salt stress. All PfCAMTA proteins contain the conserved CG-1, ANK, and IQ domains, although their gene structures and physicochemical properties differ. Phylogenetic and synteny analyses revealed close paralogous pairs and orthologous relationships with Arabidopsis CAMTAs. Under salt stress, the eight *PfCAMTA* genes displayed distinct tissue-specific expression patterns, with *PfCAMTA5* emerging as the most prominent salt-responsive candidate, showing broad induction across tissues and the highest transcript abundance. Molecular docking and Y2H assays suggested a potential interaction between PfCAMTA5 and the salt-responsive calcium sensor PfCML28. Transient overexpression of *PfCAMTA5* in tobacco improved salt tolerance, as reflected by enhanced photosynthesis, higher antioxidant enzyme activities, and reduced membrane damage, and up-regulation of stress-responsive genes. Together, these findings suggest that *PfCAMTA5* may play a potential role in salt tolerance and provide a foundation for further investigation of the CAMTA-mediated salt stress regulatory network in *P. fortunei*.

## 1. Introduction

Soil salinization is a widespread environmental constraint that severely limits the growth and productivity of woody plants. High soil salinity adversely affects plant morphology, biomass accumulation, and yield, posing a threat to sustainable forestry and production of economic forests. Salt stress suppresses plant growth and induces extensive transcriptional and metabolic reprogramming, including alterations in xylem development and cell wall composition [1,2]. Upon salt exposure, cytosolic Ca^2+^ levels increase rapidly [3,4]. The resulting Ca^2+^/CaM signals are integrated by downstream transcription factors to reprogram gene expression, constituting a central regulatory mechanism for adaptation to salt stress in plants [5,6].

CAMTA (calmodulin-binding transcription activator) proteins belong to the well characterized CaM-binding transcription factor family in plants and act as direct effectors linking Ca^2+^/CaM signaling to transcription [7,8]. CAMTA proteins contain four conserved domains: an N-terminal CG-1 domain for sequence-specific DNA binding, a TIG/IPT domain for DNA contacts and dimerization, ankyrin repeats for protein-protein interactions, and C-terminal IQ motifs for CaM binding [9]. This modular structure enables CAMTAs to integrate calcium signals and regulate target genes. Functional studies have shown that CAMTAs regulate abiotic stress responses in various plant species. In *Arabidopsis*, CAMTA1 regulates drought responses by directly targeting stress-inducible genes such as *RD26* and *ERD7* and modulating ABA signaling [10], and CAMTA6 maintains Na^+^ homeostasis during germination by directly regulating *HKT1;1* and ABA-responsive salt genes [11]. In *Fraxinus velutina*, *FvCAMTA1* is induced by salt stress, and its overexpression enhances salt tolerance in *Arabidopsis*, whereas *Arabidopsis camta5* mutants are salt-sensitive [12]. In *Populus trichocarpa*, seven *PtCAMTA* genes respond to mannitol, NaCl, cold, and phytohormones [13]. While these reports establish the involvement of CAMTAs in woody plant stress responses, most remain limited to genome-wide inventories and expression surveys; few have pursued functional characterization to connect CAMTA activity with calcium-dependent salt adaptation. Despite these limitations, available findings suggest that CAMTAs may function as upstream regulators converting Ca^2+^/CaM signals into transcriptional reprogramming in woody plants.

*Paulownia* is a woody genus with considerable salt tolerance, and considerable efforts have been made to elucidate its salt tolerance mechanisms. Numerous salt-responsive genes and miRNAs, including members of the *AP2/EREBP*, *bHLH*, *MYB*, and *NAC* transcription factor families, have been identified in *Paulownia* under salt stress [14,15]. Functional studies have further characterized *PfMYB44* and *PfMYB90* as positive regulators of salt tolerance that function by transcriptionally upregulating the SOS pathway genes *AtSOS1*, *AtSOS2*, and *AtSOS3*, as well as the Na^+^/H^+^ antiporter gene *AtNHX1* [16,17]. At the protein level, the PfCHX16–PfNHX2 module and the PfnsLTPd-5–PfGELP24 interaction have been shown to participate in ion homeostasis and stress signaling [18,19]. However, these studies have primarily focused on downstream ion homeostasis and stress signaling, whereas how salt-induced Ca^2+^/CaM signals are translated into transcriptional responses in *Paulownia* remains unknown. In particular, whether CAMTAs mediate this process remains to be determined, and no CAMTA family member has yet been functionally characterized in paulownia.

Here, we performed a genome-wide identification of the *CAMTA* gene family in *P. fortunei* and analyzed the physicochemical properties, phylogenetic relationships, conserved motifs, gene structures, and promoter *cis*-elements of these genes. We then examined their expression patterns under salt stress and identified several PfCAMTAs with significant salt-responsive expression. Among them, *PfCAMTA5* was selected as a key candidate for functional characterization. Subcellular localization and transient overexpression assays in tobacco showed that PfCAMTA5 is localized to the nucleus, and that overexpression of *PfCAMTA5* was associated with improved salt tolerance, as reflected by increased activities of antioxidant enzymes (SOD, POD, and CAT), along with reduced MDA accumulation. This study will help reveal whether *PfCAMTA5* acts as a CaM-binding transcription factor relaying salt-induced Ca^2+^ signals to downstream salt-adaptive responses, and may establish *PfCAMTA5* as a potential key salt-responsive gene laying a foundation for exploring calcium-dependent transcriptional regulation and salt tolerance mechanisms in paulownia.

## 2. Results

### 2.1. Identification and Sequence Characterization of PfCAMTA Genes

**Figure 1:**
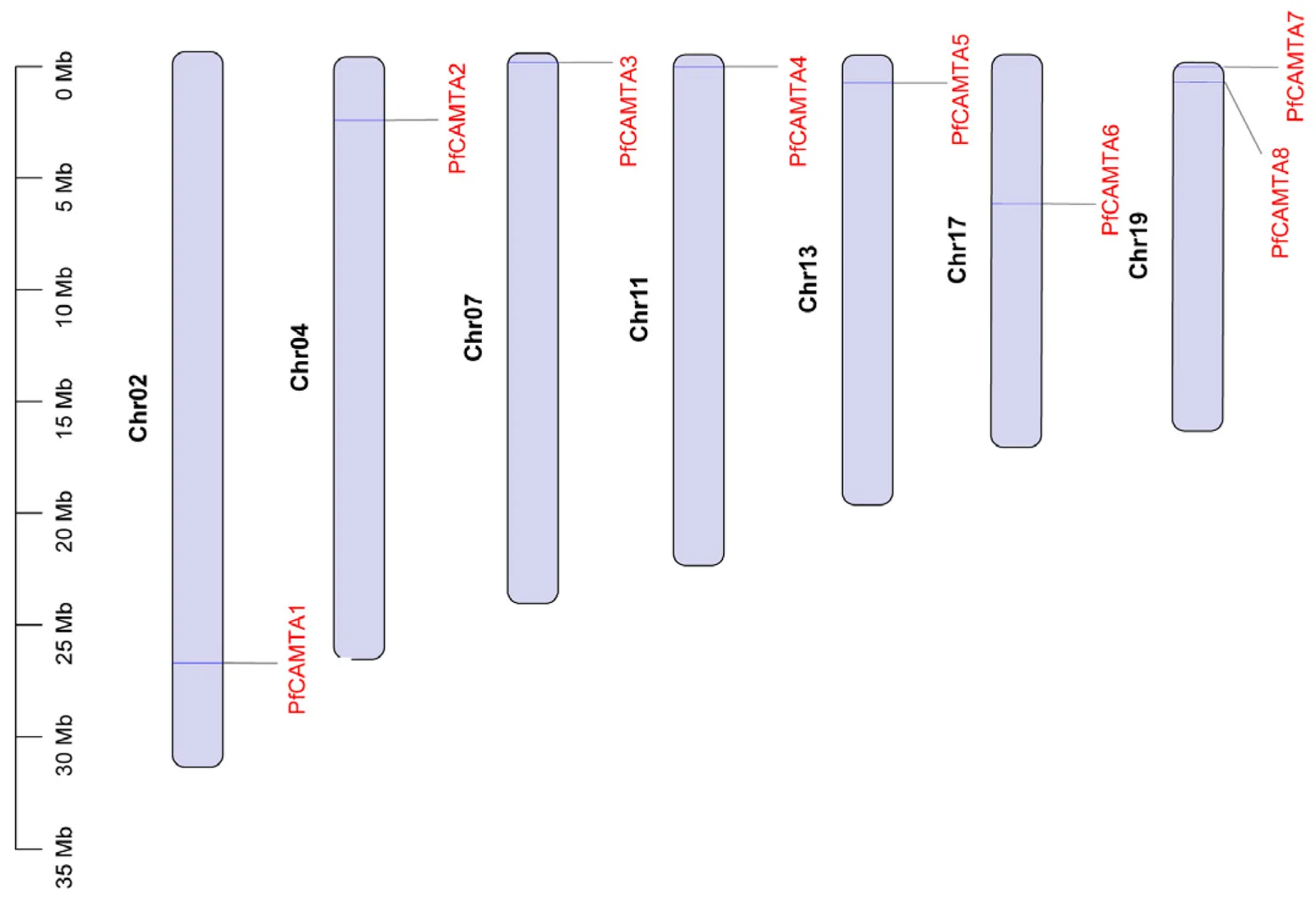
Chromosomal distribution of *PfCAMTA* genes. Chromosomal positions are denoted by black scale bars.

**Figure 2:**
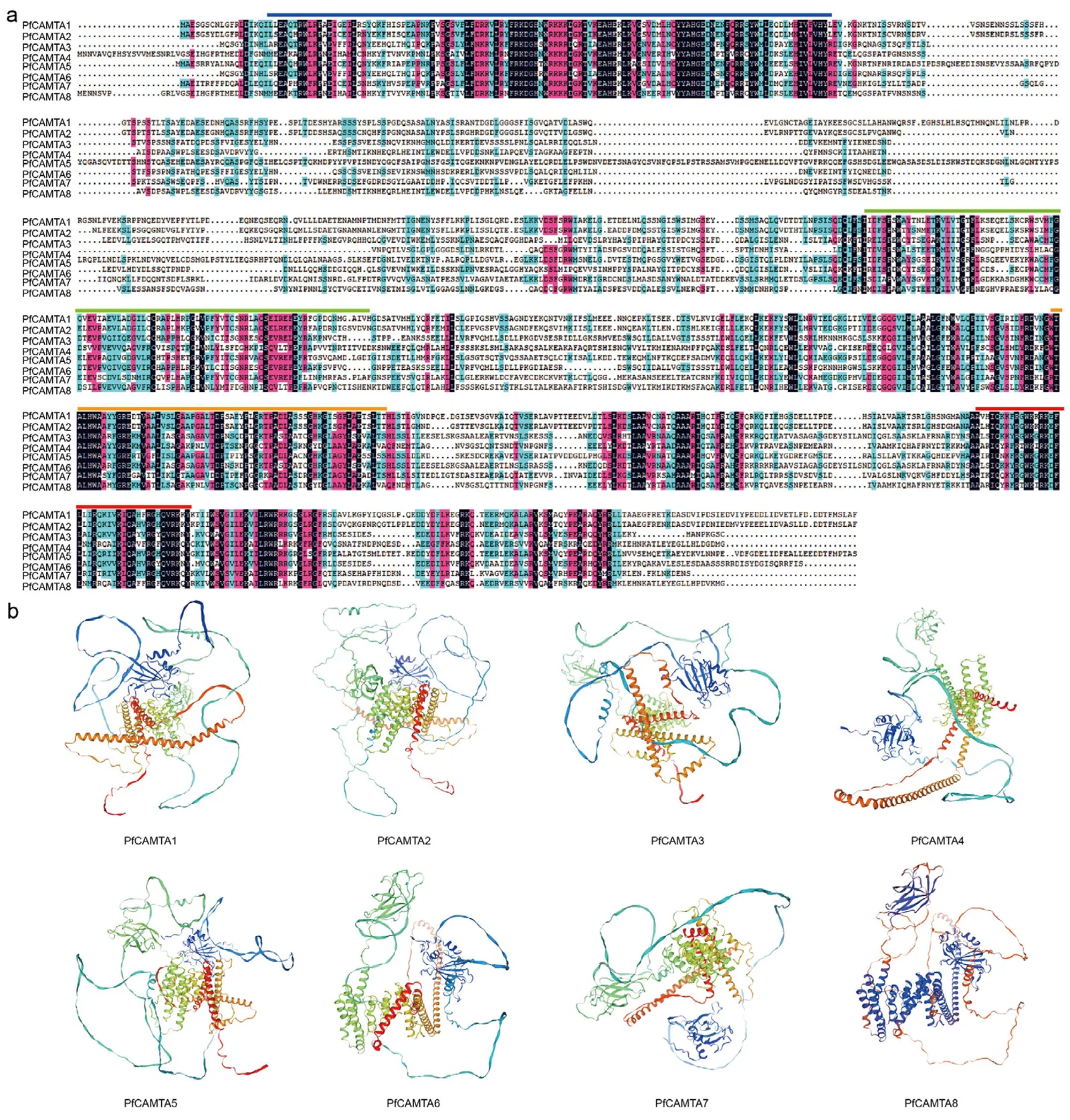
Sequence characterization of PfCAMTAs. (**a**) Conserved structural domains of the paulownia CAMTA gene family. Blue boxes are CG-1 DNA-binding structural domains, green boxes are IPT structural domains, orange boxes are ANK repeat structural domains, and red boxes are IQ motifs. (**b**) Predicted tertiary structure of PfCAMTAs.

A total of eight CAMTA family members were identified in the *P. fortunei* genome, and were named *PfCAMTA1* to *PfCAMTA8* based on their chromosomal localization (Figure 1, Table 1). Multiple sequence alignment revealed that all eight PfCAMTA proteins harbor the conserved CAMTA domains CG-1, ANK, and IQ, arranged from the N-terminus to the C-terminus (Figure 2a, Figure S1). Homology modeling further showed that these domains fold into a conserved multi-domain architecture (Figure 2b). The N-terminal region, containing the CG-1 and IPT domains, was rich in β-strands, with the CG-1 domain forming a β-sheet conformation that mediates sequence-specific DNA binding and the IPT domain displaying multiple β-strands and loop regions that primarily mediate protein–protein interactions and dimerization. The central ANK repeat region was modeled as helical bundles that provide a structural platform for protein complex assembly. At the C-terminus, the IQ motif was predicted to fold into an α-helix that mediates Ca^2+^-independent calmodulin binding.

Post-translational modification prediction revealed that all eight PfCAMTA proteins were predicated to contained phosphorylation sites, with the number ranging from 4 to 12. Among them, PfCAMTA5 had the highest number (12), followed by PfCAMTA1 and PfCAMTA2 (10 each), whereas PfCAMTA4 and PfCAMTA8 had the fewest (4 each). In addition to phosphorylation, ubiquitination sites were also predicted, with the number of ubiquitination sites was considerably lower, ranging from 0 to 2 per protein. PfCAMTA4, PfCAMTA5, and PfCAMTA7 each contained two ubiquitination sites, while PfCAMTA6 had none. Notably, PfCAMTA5 was predicted to contain the largest number of phosphorylation sites (12) as well as two ubiquitination sites (Figure S2, Table S2). These predictions provide a basis for further investigation of how post-translational modifications might affect the function of PfCAMTA proteins. Collectively, these analyses highlight the conserved structural architecture and potential post-translational modifications of PfCAMTA proteins, providing a foundation for functional characterization in *P. fortunei*.

### 2.2. Physicochemical Properties and Structural Characterization of PfCAMTA Proteins

Analysis of physicochemical properties showed that the PfCAMTA proteins ranged from 899 (PfCAMTA4) to 1114 amino acids(PfCAMTA5) in length, with corresponding molecular weights ranging from 101.31 to 125.31 kDa. The theoretical isoelectric points varied from 5.31 (PfCAMTA5) to 8.13 (PfCAMTA4), indicating that most PfCAMTA proteins are acidic, while PfCAMTA4 and PfCAMTA8 are slightly basic. The grand average of hydropathicity (GRAVY) values for all eight proteins were negative, ranging from −0.590 (PfCAMTA5) to −0.371 (PfCAMTA7), suggesting that they are predominantly hydrophilic. Subcellular localization prediction indicated that all eight PfCAMTA proteins localize to the nucleus, and PfCAMTA3, PfCAMTA4, and PfCAMTA6 were additionally predicted to localize to the cytoplasm (Table 1).

**Figure 3:**
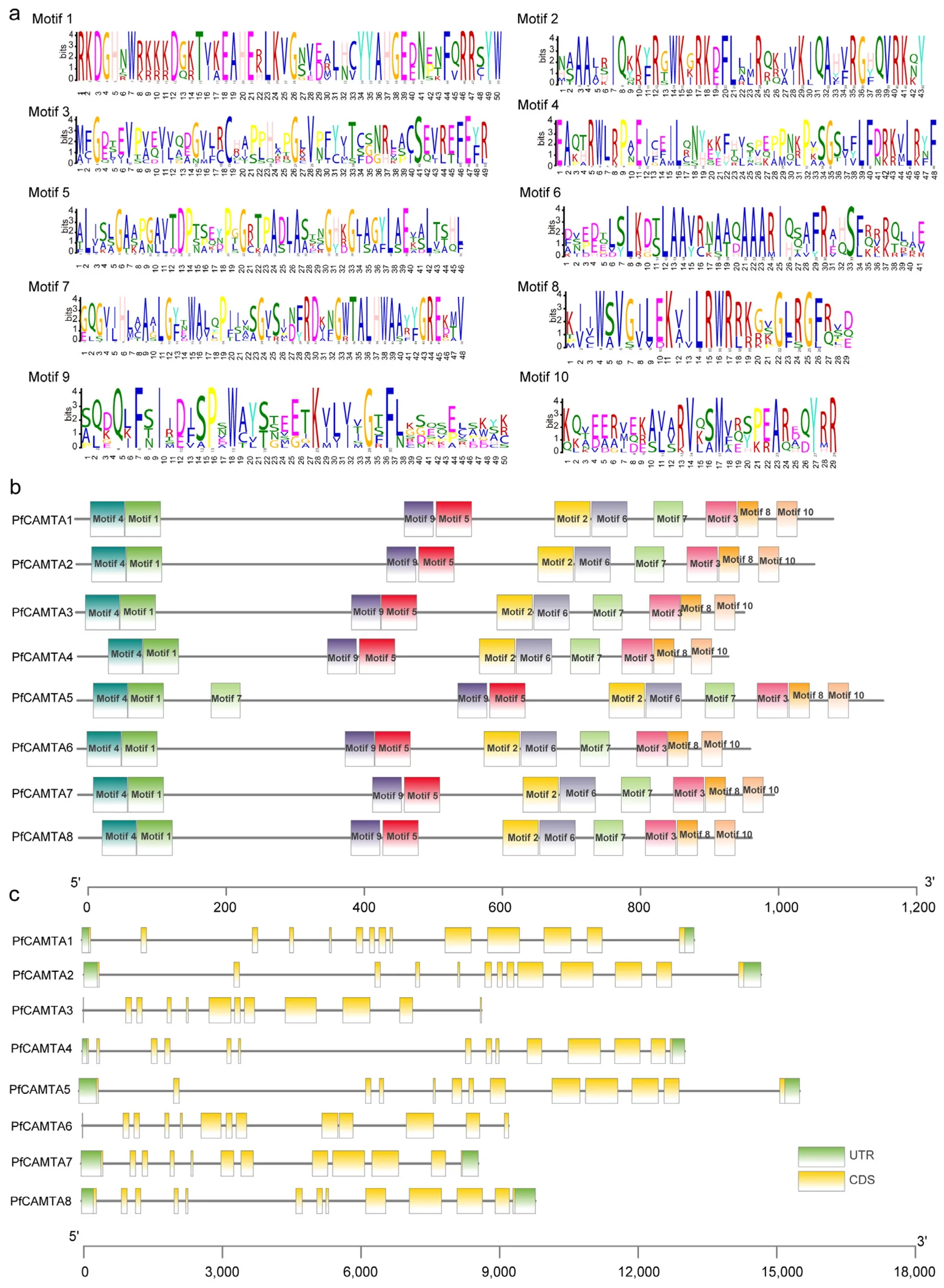
Conserved motifs and gene structures analysis of PfCAMTA proteins. (**a**,**b**) Conserved motif and gene structure analysis of paulownia CAMTA proteins. Ten conserved protein motifs (Motifs 1–10) were identified using MEME analysis (E-value < 1 × 10**^−^**^5^). Solid lines indicate protein lengths, and color-coded blocks represent individual motifs (**a**). Sequence logos show colored single-letter amino acids residues, with height proportional to frequency. Numbers below each logo indicate motif length, and bit scores on the left indicate conservation level (**b**). (**c**) Exon-intron architecture of *PfCAMTAs*. Exon-intron architecture was visualized with TBtools. Green blocks represent the untranslated regions, yellow blocks represent coding sequences, and line segments represent introns.

Conserved motif analysis showed that these motifs were primarily composed of hydrophobic (A, V, L, I, F, M), charged (E, D, R, K), and polar (S, T, N, Q) residues (Figure 3a), suggesting that they may mediate protein–protein interactions and contribute to nuclear functions such as DNA binding and transcriptional regulation. Motif distribution analysis revealed that all eight PfCAMTA proteins shared a common set of ten motifs (Motif 1–10). Seven of them exhibited a highly similar motif order, whereas PfCAMTA5 contained an additional N-terminal copy of motif 7 (Figure 3b). These results suggest that the motif composition and arrangement are conserved across most *PfCAMTA* family members. To further examine the gene structures of the *PfCAMTA* genes, their exon–intron organization was analyzed (Figure 3c). The number of exons ranged from 12 to 14 among the eight genes, and the exon lengths also varied among the family members. These results indicate that although the PfCAMTA family is conserved in motif composition and arrangement, the gene structures exhibit some variation.

### 2.3. Phylogenetic Relationships and Cis-Regulatory Element Profiles of PfCAMTA Genes

**Figure 4:**
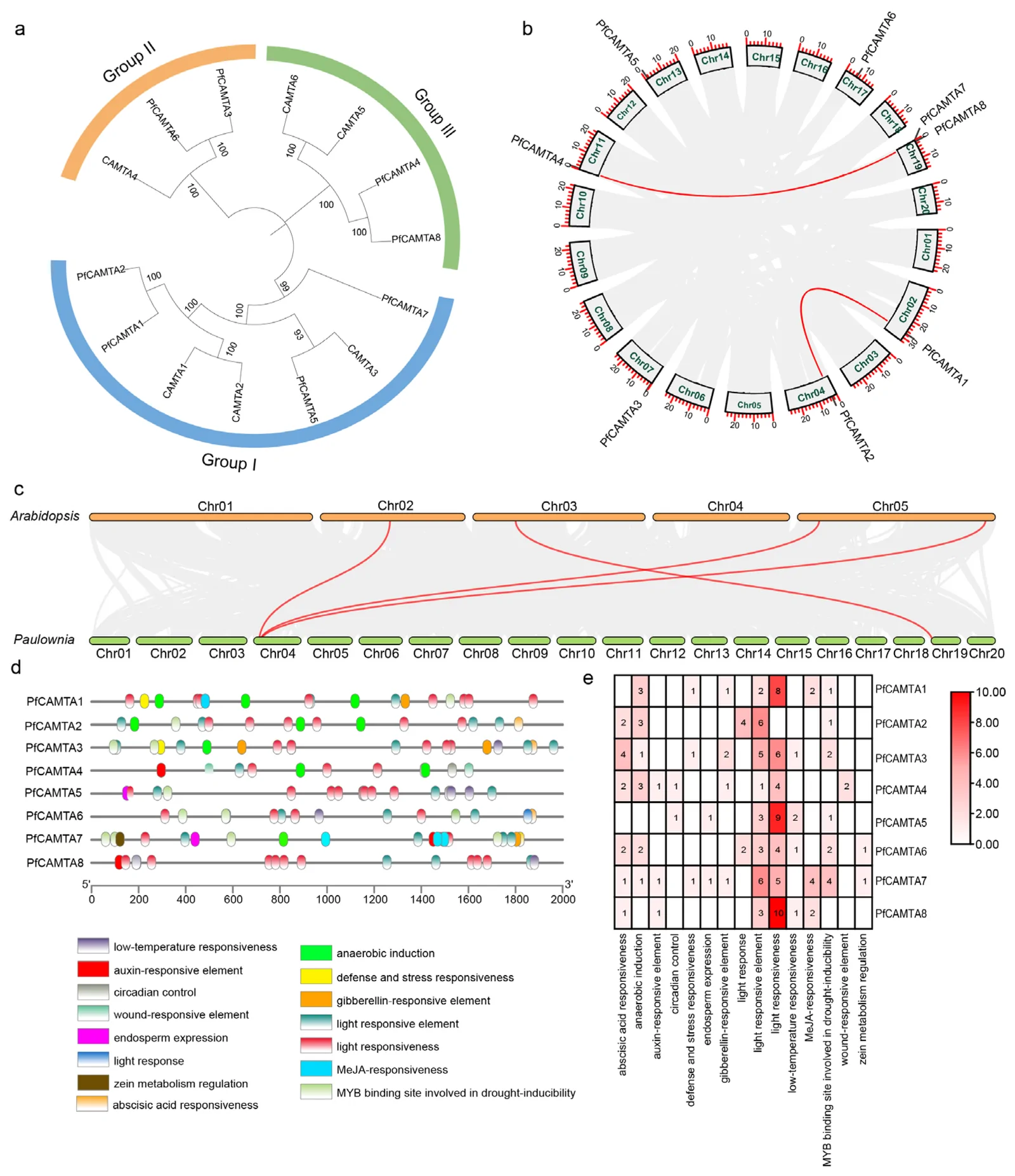
Phylogenetic tree and Synteny analysis of *PfCAMTA* genes. (**a**) Phylogenetic analysis of PfCAMTA proteins in *Paulownia* and *Arabidopsis*. The tree revealed three CAMTA subfamilies, and bootstrap support values from 1000 replicates are shown at branch nodes. (**b**) Intra-genomic collinearity of the *PfCAMTA* genes. (**c**) Synteny analysis showing the evolutionary relationships between *CAMTA* genes in *P. fortunei* and *A. thaliana*. Green and orange blocks represent the chromosomes of *Paulownia* and *Arabidopsis*, respectively. The red lines represent orthologous *CAMTA* pairs between the two species. (**d**) *Cis*-regulatory element distribution *in PfCAMTA* promoters. Promoter regions (2000 bp upstream) of *PfCAMTA* genes were screened using the PlantCARE database. (**e**) Distribution of the 14 *cis*-regulatory elements among the *PfCAMTAs* genes Promoter, the numbers indicate the quantity of *cis*-acting elements.

The phylogenetic tree illustrates the evolutionary relationships among CAMTA proteins in *P. fortunei*. The topology reveals that these PfCAMTA proteins cluster into three distinct clades, supported by bootstrap values ranging from 93% to 100% (Figure 4a). Notably, genes located on different chromosomes, such as *PfCAMTA1*/*PfCAMTA2* and *PfCAMTA4*/*PfCAMTA8*, form closely related paralogous pairs in the tree, and these pairs are located within syntenic blocks (Figure 4b). Synteny analysis revealed a total of four orthologous blocks between *P. fortunei* and *A. thaliana* (Figure 4c). These blocks display both one-to-one (e.g., *PfCAMTA8*/*AtCAMTA6*) and one-to-many (e.g., *PfCAMTA2* with *AtCAMTA1/2/3*) orthologous relationships. Collectively, these findings are consistent with a possible contribution of gene duplication to shaping the *CAMTA* family in *P. fortunei*.

*Cis*-regulatory element analysis showed that the promoters of all eight *PfCAMTA* genes are predominantly enriched in light-responsive elements, along with multiple stress-related elements, including ABA-responsive elements (ABRE), MYB binding sites (MRE, MBS), defense and stress-responsive elements (TC-rich repeats) (Figure 4d,e, Table S3). These stress-related elements are distributed unevenly among the members, with *PfCAMTA3* and *PfCAMTA7* harboring relatively higher densities of these elements, whereas other members possess fewer. Given the pivotal roles of ABA and MeJA in coordinating stomatal behavior, oxidative stress mitigation, and photosynthetic performance, these *PfCAMTA* members are likely to be involved in salt stress responses, providing a basis for further functional characterization.

### 2.4. Expression Profiling of PfCAMTAs Under Salt Stress

**Figure 5:**
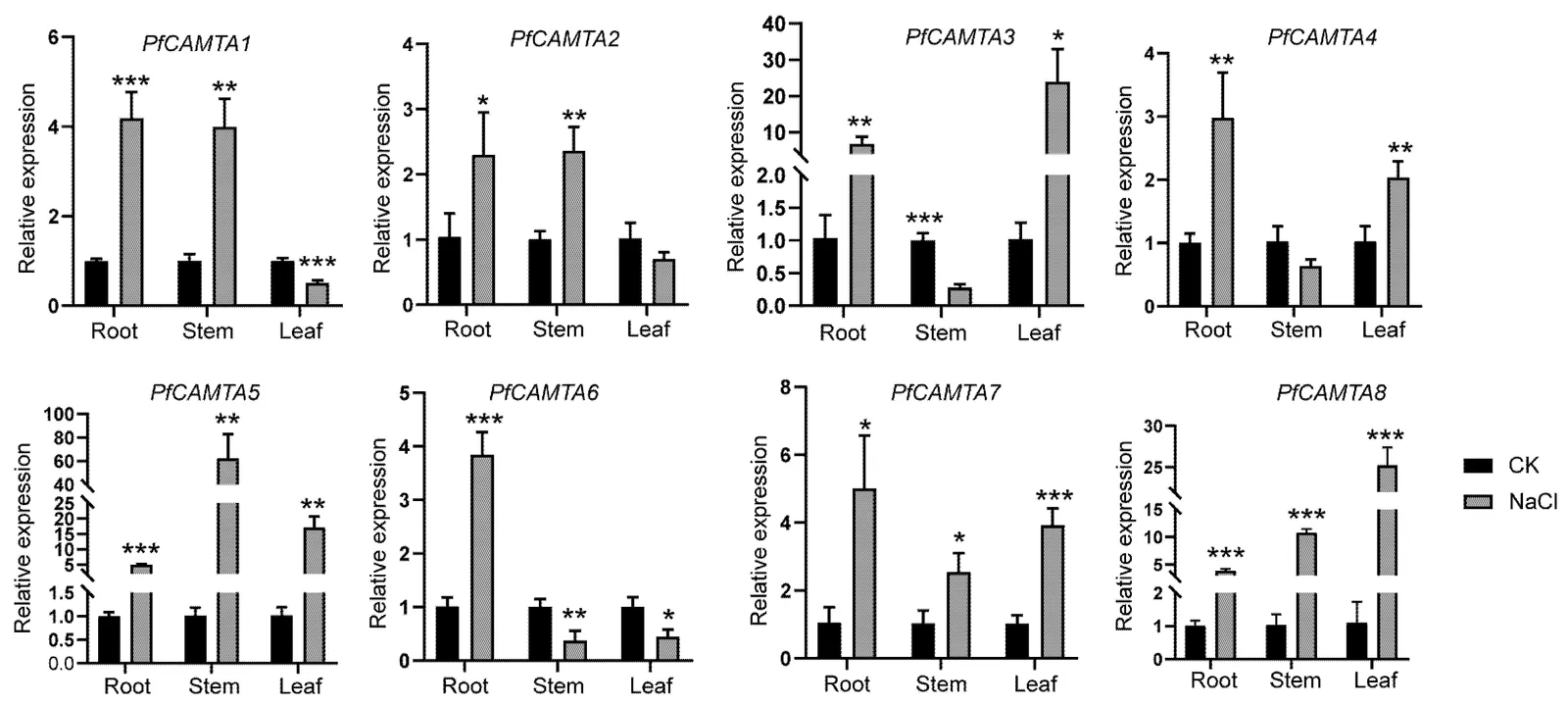
Expression analysis of *PfCAMTA* genes under salt stress. RT-qPCR analysis of *PfCAMTAs* in three tissues under salt stress. Data are presented as means ± SD from three biological replicates, and statistical differences between CK and NaCl treatment were analyzed using a two-tailed unpaired *t*-test (* *p* < 0.05, ** *p* < 0.01, *** *p* < 0.001).

To investigate the expression patterns of *PfCAMTA* genes under salt stress, all eight members were examined by RT-qPCR in roots, stems, and leaves, and their relative expression levels were calculated. Distinct tissue-specific expression patterns were observed (Figure 5). *PfCAMTA3* and *PfCAMTA8* were predominantly up-regulated in leaves; *PfCAMTA1* and *PfCAMTA2* were preferentially induced in roots and stems; *PfCAMTA4* was mainly induced in roots and leaves; *PfCAMTA6* was predominantly induced in roots; and *PfCAMTA5* and *PfCAMTA7* were broadly up-regulated across all three tissues, with *PfCAMTA5* showing the strongest induction in stems and *PfCAMTA7* displaying relatively uniform induction in roots, stems, and leaves.

Compared with the control, these members exhibited distinct salt-responsive fold changes in a tissue-dependent manner. Specifically, *PfCAMTA3* and *PfCAMTA8* exhibited the most pronounced up-regulation in leaves, with approximately 24-and 25-fold increases, respectively. In contrast, *PfCAMTA6* displayed strong root-specific induction (up to 4-fold) but was significantly down-regulated in stems and leaves (reduced to approximately 0.3-fold and 0.4-fold, respectively). Interestingly, *PfCAMTA4* displayed moderate induction in roots and leaves (approximately 3- and 2-fold, respectively) and slight repression in stems (approximately 0.6-fold). Similarly, *PfCAMTA1* and *PfCAMTA2* were significantly induced in roots and stems (approximately 4-fold and 2.3-fold), but remained minimally responsive in leaves. Meanwhile, *PfCAMTA7* was consistently up-regulated across all three tissues (approximately 5-fold in roots; 2.5-fold in stems; 4-fold in leaves). While these genes exhibited distinct tissue-specific responses, *PfCAMTA5* emerged as the most prominent and broad-spectrum salt-responsive candidate. Under salt stress, *PfCAMTA5* was significantly induced across all three tissues, with approximately 5-fold up-regulation in roots, 60-fold induction in stems, and 15-fold up-regulation in leaves, reaching the highest transcript abundance among all tested genes (Figure 5). In addition, PfCAMTA5 was predicted to contain the largest number of phosphorylation sites (12) and two ubiquitination sites among the PfCAMTA family members. Based on these expression and structural features, PfCAMTA5 was selected for further functional characterization. Furthermore, promoter analysis identified multiple stress-related *cis*-acting elements in *PfCAMTA* genes, providing candidate regulatory motifs for further investigation of their salt-responsive expression.

### 2.5. Expression Analysis of PfCMLs and Prediction of Potential PfCAMTA5-CML Interactions Under Salt Stress

**Figure 6:**
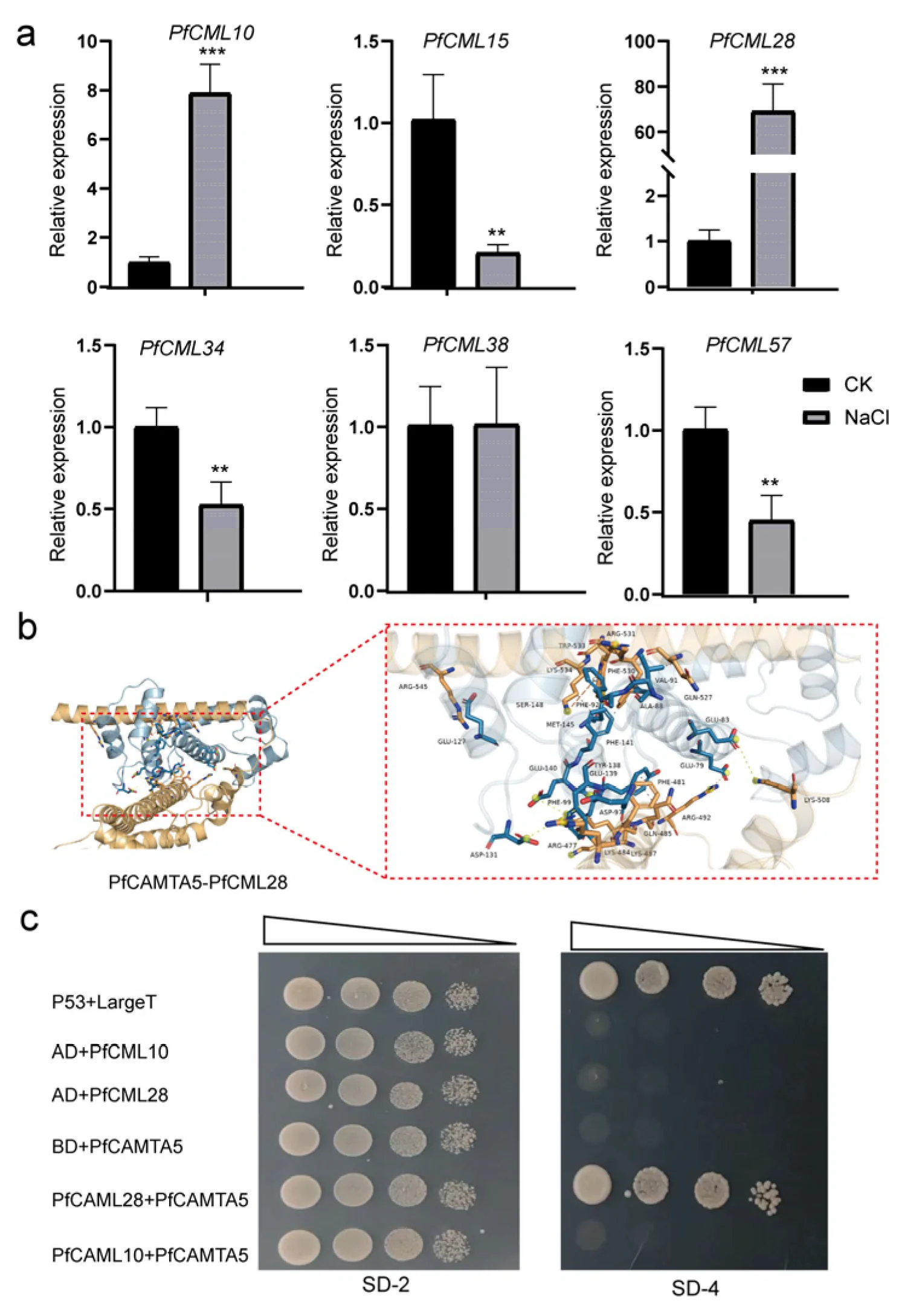
Analysis of PfCAMTA5-PfCMLs interactions. (**a**) Expression analysis of *PfCMLs* under salt stress. Values are means ± SD from three biological replicates, and statistical differences between CK and NaCl treatment were analyzed using a two-tailed unpaired *t*-test (** *p* < 0.01, *** *p* < 0.001). (**b**) Molecular docking simulation between PfCAMTA5 and PfCMLs predicted key interaction patterns at the binding interface. Protein structures were built using SWISS-MODEL, and the docking conformation were calculated using ZDOCK. (**c**) In vivo interaction between PfCAMTA5 and PfCMLs by the Y2H. SD-2, minimal media without Leu and Trp; SD-4, minimal media without Leu, Trp, Ade, and His. The combinations of AD + PfCML10, AD + PfCML28, and BD + PfCAMTA5 served as negative controls to exclude autoactivation, and P53+LargeT served as a positive control. Photographs were taken after 3 to 5 d.

Calcium acts as a second messenger in salt-stress signaling. CAMTA transcription factors contain a C-terminal IQ motif that mediates CaM/CML binding. To investigate whether CMLs participate in CAMTA-mediated salt stress responses, we examined the expression of six previously identified Ca^2+^-responsive *PfCML* genes under salt treatment [20]. Among them, five *PfCML* genes exhibited significant transcription induction or repression under salt stress. *PfCML10* and *PfCML28* were up-regulated by approximately 8- and 70-fold, respectively, whereas *PfCML15*, *PfCML34*, and *PfCML57* were down-regulated by about 5-, 2-fold, and 3-fold, respectively (*p* < 0.05) (Figure 6a). Given the extent of these expression changes and their similar expression trends to *PfCAMTA5*, *PfCML10* and *PfCML28* were selected as candidates for further interaction assays with *PfCAMTA5*. To this end, molecular docking was performed to predict the potential binding modes between PfCAMTA5 and these two candidate PfCMLs. The docking analysis revealed that PfCAMTA5 showed a strong predicted binding affinity for PfCML28 (binding energy = −13.8 kcal/mol), whereas the predicted binding energy for PfCML10 was far weaker (−1.7 kcal/mol) (Table S4). The predicted PfCAMTA5–PfCML28 complexes were primarily stabilized by hydrophobic interactions, π-cation interactions, and salt bridges (Figure 6b, Table S4). Interface analysis of the PfCAMTA5–PfCML28 complex identified was mainly stabilizaed by 11 hydrophobic interactions, 2 π-cation interactions, and 7 salt bridges (Table S4). In this model, residues Glu79, Glu83, Asp97, Glu127, Asp131, Glu139, and Glu140 formed salt bridges with PfCML28 at distances ranging from 3.41 to 5.41 Å. Comparable hydrophobic interactions, π-cation interactions, and salt bridges networks were also observed in the PfCAMTA5–PfCML10 complex (Table S4). Notably, the predicted binding interface between PfCAMTA5 and PfCMLs was located in the C-terminal IQ motif of PfCAMTA5. To experimentally validate these predictions, yeast two-hybrid assays were performed. Consistent with the docking results, PfCAMTA5 interacted with PfCML28, but no detectable interaction was observed between PfCAMTA5 and PfCML10 (Figure 6c). These results provide preliminary evidence that PfCAMTA5 may interact with PfCML28.

### 2.6. Transient Overexpression of PfCAMTA5 Enhanced Salt Tolerance in Tobacco

**Figure 7:**
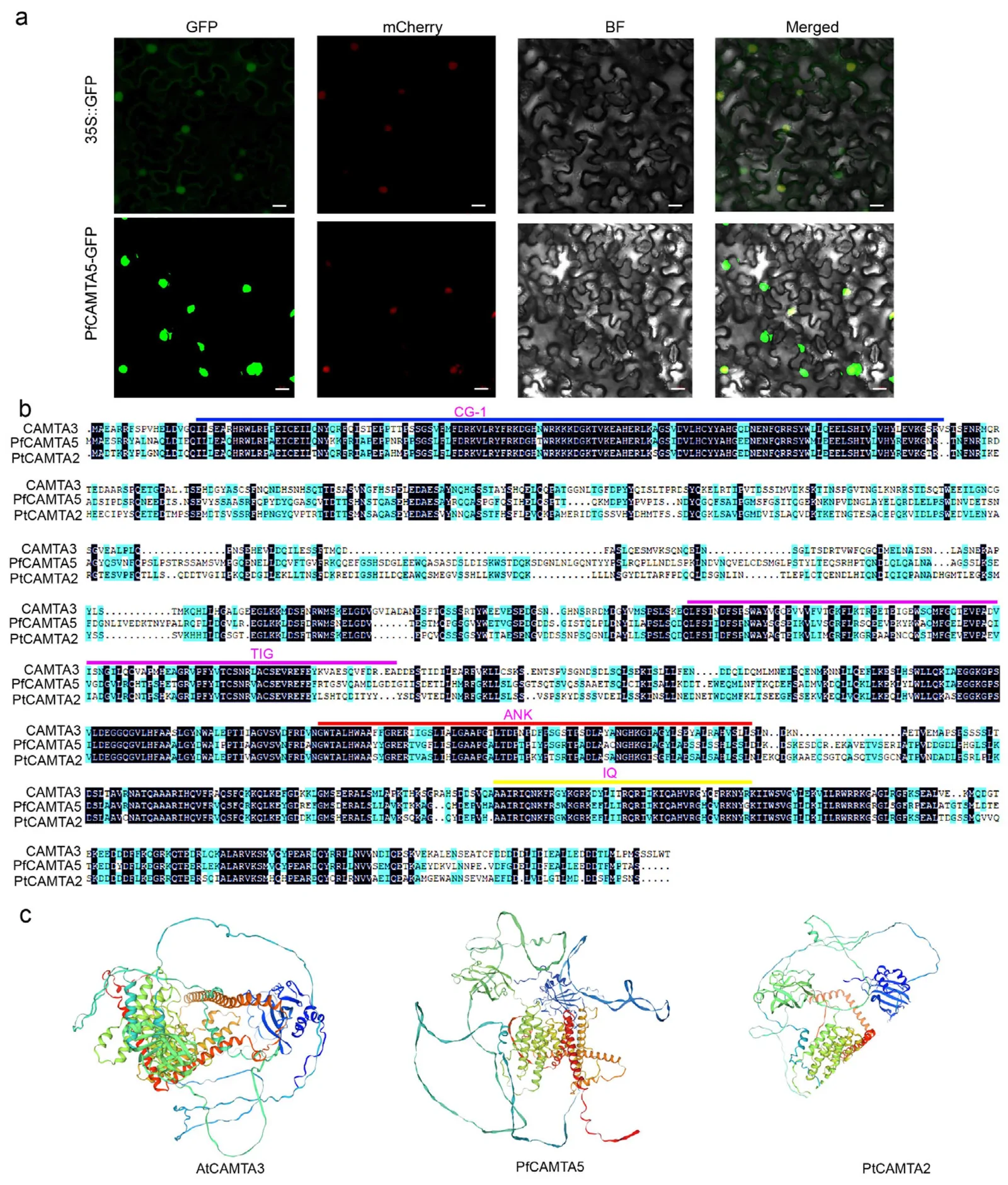
Subcellular localization analysis of PfCAMTA5. (**a**) Subcellular localization of PfCAMTA5. The *PfCAMTA5*-GFP fusion protein was transiently overexpressed in *N. benthamiana* epidermal cells. AtH2B was used as marker. Bar = 20 μm (**b**) Alignment of amino acid sequences of CAMTA from *A. thaliana*, *P. fortunei*, and *P. trichocarpa*. DNAMAN was used for multiple sequence alignment and visualization. Amino acid residues shaded in black represent identical residues. (**c**) Comparative three-dimensional (3D) structural analysis of CAMTA proteins. Homology models of AtCAMTA3, PtCAMTA2 and PfCAMTA5 were built via SWISS-MODEL.

To investigate the role of *PfCAMTA5* in salt stress responses, its full-length coding sequence (CDS) was cloned from *P. fortunei*. The CDS encodes a polypeptide of 1114 amino acids with a predicted molecular weight of 125 kDa and a theoretical isoelectric point of 5.3. Subcellular localization prediction indicated that PfCAMTA5 was predominantly localized in the nucleus, consistent with its function as a transcription factor (Figure 7a). Sequence alignment showed that PfCAMTA5 shares high similarity with CAMTA proteins from *A. thaliana* and *P. trichocarpa*, and harbors the typical conserved CAMTA domains (Figure 7b,c).

**Figure 8:**
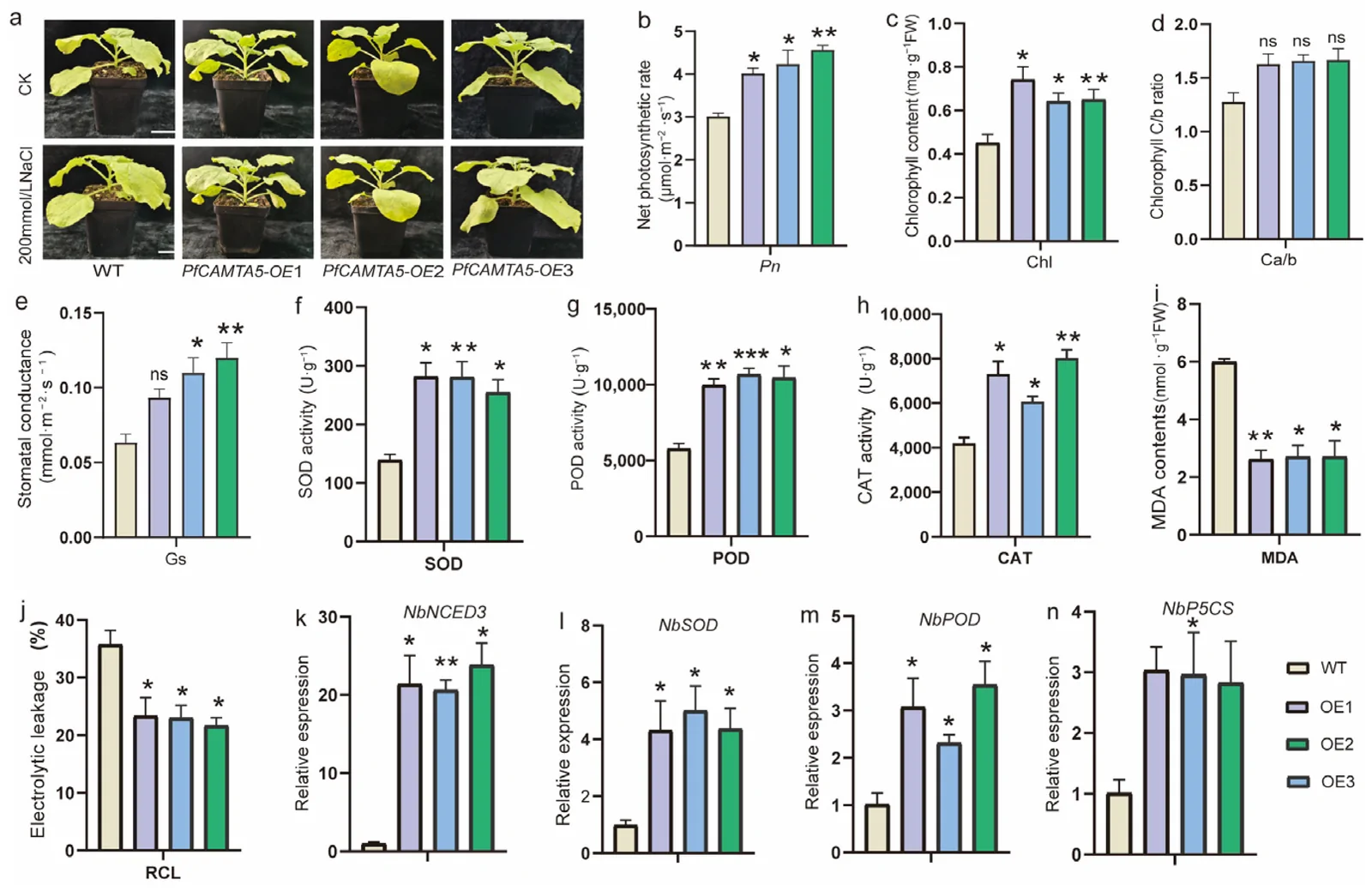
*PfCAMTA5* over-expression enhances salt stress tolerance in tobacco. (**a**) Phenotypic comparison between EV and *PfCAMTA5*-OE plants (OE1, OE2, and OE3) under control and salt stress conditions. EV, leaves infiltrated with the empty vector pRI101-GFP; OE1, OE2, and OE3 three independent transiently transformed tobacco plants overexpressing *PfCAMTA5*-GFP. Bar = 2 cm. (**b**–**e**) Net photosynthetic rate (**b**), total chlorophyll content (**c**), chlorophyll a/b ratio (**d**), and stomatal conductance (**e**) in EV and three independent *PfCAMTA5* transiently overexpressing plants under salt stress. (**f**–**i**) Activities of antioxidant enzymes SOD (**f**), POD (**g**), and CAT (**h**), and MDA content (**i**) in EV and *PfCAMTA5* transiently overexpressing plants under salt stress. (**j**) Relative electrolyte leakage in EV and *PfCAMTA5* transiently overexpressing plants under salt stress. (**k**–**n**) Relative transcript levels of stress-responsive genes *NbNCED3* (**k**), *NbSOD* (**l**), *NbPOD* (**m**), and *NbP5CS* (**n**) in EV and *PfCAMTA5* transiently overexpressing plants under salt stress, as determined by RT-qPCR. Data are presented as means ± SD of three biological replicates. Asterisks indicate significant differences between EV and *PfCAMTA5* transiently overexpressing plants, as determined by one-way ANOVA followed by Dunnett’s multiple comparisons test against the EV control (* *p* < 0.05, ** *p* < 0.01, *** *p* < 0.001), “ns” indicates not significant (*p* > 0.05).

To examine its function under salt stress, *PfCAMTA5* was transiently over-expressed in tobacco, with leaves infiltrated with the empty vector pRI101-GFP used as the control (EV). RT-qPCR analysis confirmed that *PfCAMTA5* was significantly up-regulated in transiently transformed leaves compared with EV (Figure S3). Phenotypic analysis showed no obvious differences between *PfCAMTA5* transiently overexpressing tobacco leaves (PfCAMTA5-OE) and EV leaves under NaCl treatment (Figure 8a). However, under salt stress, *PfCAMTA5* transiently overexpressing leaves exhibited significantly higher net photosynthetic rate, chlorophyll content, and stomatal conductance than the EV control (Figure 8b–e). In contrast, the chlorophyll a/b ratio showed only an increasing trend in *PfCAMTA5*-OE plants compared with EV under salt stress, and the differences were not statistically significant (*p* = 0.0536 and 0.0763 for two independent transiently transformed plants OE1 and OE2, respectively). In addition, the activities of antioxidant enzymes, including SOD, POD, and CAT, were markedly higher in *PfCAMTA5* over-expression plants, whereas MDA content was lower (Figure 8f–i). Moreover, relative electrolyte leakage was markedly reduced (Figure 8j) in the *PfCAMTA5* transiently overexpressing plants, suggesting improved membrane integrity and reduced oxidative damage. Consistent with these physiological improvements, RT-qPCR analysis revealed that the transcript levels of key stress-responsive genes, including *NbNCED3*, *NbSOD*, *NbPOD*, and *NbP5CS* were up-regulated in *PfCAMTA5* transiently overexpressing plants compared with the EV control, with statistically significant changes observed for these genes in at least one over-expression plant (Figure 8k–n). Collectively, these results suggest that *PfCAMTA5* may be associated with enhanced salt stress tolerance, potentially through the antioxidant defense system and maintenance of cellular membrane integrity. These findings provide a basis for further investigation of the CAMTA-mediated salt stress regulatory network in *P. fortunei*.

## 3. Discussion

CAMTA transcription factors are conserved regulators of calcium-mediated stress signaling in plants [21,22]. Members of this family have been functionally characterized across plant species, and several CAMTA genes have been implicated in salt stress responses [23,24]. In this study, eight *CAMTA* genes were identified in the *P. fortunei* genome, and *PfCAMTA5* was characterized as a positive regulator of salt tolerance. The results expand our understanding of the *CAMTA* gene family in woody plants and provide a basis for further functional studies and molecular breeding of new paulownia varieties.

The PfCAMTA family members displayed both structural conservation and divergence. All eight PfCAMTA proteins shared a common set of ten motifs (Motif 1–10), and seven of them exhibited a highly similar motif order. However, PfCAMTA5 was an exception, harboring an additional N-terminal copy of motif 7 beyond the shared ten motifs. This indicates that motif composition is largely, but not entirely, conserved within the family. Whether this additional motif copy contributes to functional divergence remains to be experimentally determined. Exon–intron analysis further revealed structural variation, with exon numbers ranging from 12 to 14 among the eight genes. Members within the same phylogenetic clade tended to share similar exon distribution patterns, even though exon numbers varied slightly among them, suggesting that structural features are associated with phylogenetic relationships. In addition, members of the *PfCAMTA* family with closer evolutionary relationships had similar PTM numbers and locations, exhibiting relative conservation. Together, these results indicate that although the *PfCAMTA* family is conserved in motif composition and overall gene structure, structural divergence has occurred in specific members, which may contribute to functional diversification within the family. Functional characterization of representative members from each clade will be required to test this possibility.

*PfCAMTA* genes exhibit functional diversity under salt stress. Previous studies have shown that CAMTA family members play distinct roles in salt-stress signaling, including promoting root growth and maintaining ROS homeostasis [11,12,25]. Under salt stress, the eight *PfCAMTA* genes were not uniformly induced but instead displayed distinct tissue-preferential patterns: (1) Promoter architecture. *PfCAMTA* promoters are enriched with light-responsive elements and multiple stress-related *cis*-acting elements, including ABRE, MRE, TCA-element, TGACG-motif, and MBS. The uneven distribution of these elements among *PfCAMTA* genes, with *PfCAMTA3* and *PfCAMTA8* harboring relatively higher densities, is consistent with the stronger induction of these two genes in leaves, although functional validation is required to establish a causal relationship. However, *PfCAMTA5* reached the highest transcript abundance despite not having the highest density of stress-related elements, suggesting that additional regulatory layers, such as chromatin accessibility, enhancer–promoter interactions, or post-translational modification, may also be involved, although these possibilities remain to be experimentally validated. (2) Tissue-specific expression. *PfCAMTA6* showed root-specific induction; *PfCAMTA1*, *PfCAMTA2*, and *PfCAMTA4* were moderately induced in roots; *PfCAMTA3* and *PfCAMTA8* were strongly induced in leaves; and *PfCAMTA5* was induced in stems, roots, and leaves, with the strongest induction in stems. These distinct expression patterns indicate that *PfCAMTA* genes are differentially regulated across organs under salt stress.

*PfCAMTA5* may function as a central regulator of salt tolerance in *P. fortunei*. Among the eight *PfCAMTA* members, *PfCAMTA5* exhibited the highest transcript abundance, the most pronounced salt-responsive induction across tissues, and the greatest number of predicted post-translational modification sites. In *Arabidopsis*, CAMTA3 is phosphorylated by MPK3/MPK6, which promotes its nuclear export and destabilization [26]. These findings indicate that phosphorylation can regulate CAMTA protein function. Similarly, the predicted phosphorylation sites on PfCAMTA5 suggest that it may undergo post-translational regulation under salt stress, enabling fine-tuning without altering transcript levels. In addition to phosphorylation, CAMTA activity is regulated by calcium sensors, including both CaM and CML proteins. In *Arabidopsis*, CML13 and CML14 bind the IQ domains of all six CAMTAs, and *cml13* mutants show enhanced salinity tolerance during germination, similar to *camta6* mutants, with altered expression of salt stress-related marker genes [27]. In rice, the OsCaM1-OsCAMTA1 module enhances salt tolerance by activating *OsDREB1B* and *OsCML16*, with *OsCAMTA1* and *OsCML16* co-regulating *OsP5CS* and other downstream genes [28]. In the present study, *PfCML28* was strongly induced by salt stress. Docking predicted a possible interaction between PfCAMTA5 and PfCML28, and Y2H assays provided preliminary support for this prediction. Since CAMTA proteins typically bind CaM/CML through their C-terminal IQ motif, we further examined whether the predicted interface overlapped with this region. The interface residues were located inside the IQ motif of PfCAMTA5, suggesting a possible canonical binding mode. These findings suggest a potential link between calcium signals and PfCAMTA5-mediated transcription. Consistent with a potential role for *PfCAMTA5* in salt tolerance, transient overexpression of *PfCAMTA5* in tobacco was associated with improved salt tolerance, accompanied by up-regulation of *NbNCED3*, *NbP5CS*, *NbPOD*, and *NbSOD*, as well as improved photosynthesis, stomatal behavior, antioxidant activity, and membrane integrity. However, we note that the physiological phenotypes detected in *PfCAMTA5*-overexpressing tobacco may partly arise from secondary adaptive responses following stress, and ROS quantification or histochemical staining (DAB/NBT for H_2_O_2_ and O_2_^−^) are needed to solidify the oxidative homeostasis regulatory model. Given that only NaCl treatment was applied, we cannot distinguish whether these physiological improvements are caused by enhanced tolerance to ionic stress, osmotic stress, or a combination of both. Based on our previous PfCHX16-PfNHX2 result in *P. fortunei*, future iso-osmotic mannitol and Na^+^/K^+^ assays will help distinguish these two stress components. In addition, the present study is also limited by the experimental system. Stable genetic transformation in *P. fortunei* has not yet been established, and the PfCAMTA5-PfCML28 interaction was only validated in yeast; further validation in planta is needed. Furthermore, the direct target genes of PfCAMTA5 remain unknown. Future studies using stable transgenic lines, protein–protein interaction assays, and target identification (Y1H, EMSA, and ChIP-qPCR) will be needed to confirm the proposed mechanism.

## 4. Materials and Methods

### 4.1. Plant Material and Salt Treatment

The *P. fortunei* materials were obtained from the Paulownia Biology Laboratory of Henan Agricultural University, Zhengzhou, China. Seedlings were grown in nutrient soil for approximately 50 days under controlled conditions (25 ± 2 °C, 16 h/8 h light/dark photoperiod, 130 µmol·m^−2^·s^−1^). For salt treatment, seedlings were treated with 0.4% NaCl for 24 h as previously described [18], while control plants were treated with water. Each treatment included four biological replicates.

### 4.2. Identification and Bioinformatic Analyses of Putative PfCAMTA Genes

Genomic sequences and annotation files for *A. thaliana* were obtained from the Ensembl Plants database (https://plants.ensembl.org/index.html, accessed on 15 September 2026). The Hidden Markov Model (HMM) profiles for the CG-1 (PF03859), ankyrin (PF00023), and IQ (PF12796) domains were retrieved from the Pfam database. To identify the PfCAMTA members in the *P. fortunei* genome [29], we combined BLASTP searches (E-value < 1 × 10^−5^, coverage ≥ 50%, identity ≥ 40%) and HMMER searches (E-value < 1 × 10^−5^) based on the refined Arabidopsis CAMTA dataset from TAIR. Candidate genes were further validated using NCBI Conserved Domain Database (CDD) and SMART to confirm the domain integrity. ExPASy ProtParam server (https://web.expasy.org/protparam/, accessed on 15 September 2026) and Wolf PSORT (https://www.genscript.com/wolf-psort.html, accessed on 15 September 2026) were used to analyze Physicochemical properties and predict subcellular localization were, respectively.

To infer the phylogenetic relationships of CAMTA proteins from *P. fortunei* and *A. thaliana*, multiple sequence alignment was performed using MAFFT (v7.407) [30] and trimmed with trimAl (v1.4.rev22) [31]. A maximum-likelihood phylogenetic tree was constructed using IQ-TREE (v3.0.1) [32], with the best-fit substitution model selected by ModelFinder [33]. Bootstrap support was estimated with 1000 replicates to ensure reliability. The resulting tree was visualized and annotated using iTOL.

The chromosomal locations and gene structures of the identified *PfCAMTA* genes were extracted from the GFF file of *P. fortunei* genome and visualized using TBtools (v 2.012) [34]. MEME was used to identify conserved motifs of PfCAMTAs, with parameters set as follows: number of motifs = 10, width range of motif = 6–50 bp, and E-value < 0.05 [35].

Inter-species syntenic relationships of CAMTAs between *P. fortunei* and *A. thaliana* were identified using the MCScanX algorithm with default parameters (MATCH_SIZE = 5, E-value < 1 × 10^−5^) and visualized using the Dual Synteny Plotter in TBtools [36]. The promoter regions (~2000 base pairs) upstream of the start codons of *PfCAMTA* genes were analyzed for *cis*-regulatory elements using PlantCARE [37] (https://ngdc.cncb.ac.cn/databasecommons/database/id/5261, accessed on 15 September 2026). The tertiary structure was predicted using SWISS-MODEL (https://swissmodel.expasy.org/, accessed on 15 September 2026).

### 4.3. Post-Translational Modification Site Analysis of PfCAMTAs

The phosphorylation and ubiquitination-modification sites of PfCAMTAs were predicted with default parameters using PhytoMark (https://www.novopro.cn/tools/phytomark.html, accessed on 15 September 2026), respectively.

### 4.4. Expression Analysis by RT-qPCR

For expression analysis under salt stress, leaves, roots, and stems organs were collected from *P. fortunei* seedlings after 24 h of salt treatment. Four biological replicates were prepared for the salt treatment as described above, and three of them were used for RT-qPCR analysis. For each biological replicate, total RNA was extracted from a pooled sample of four plantlets using an RNA Extraction Kit (Vazyme, Wuhan, China). cDNA was synthesized using StarScript III One-Step RT-qPCR SYBR Kit (GenStar, Beijing, China). RT-qPCR was performed on a CFX96 Touch™ system (Bio-Rad, Hercules, CA, USA) using gene-specific primers. The cycling conditions were as follows: initial denaturation at 95 °C for 30 s, followed by 40 cycles of 95 °C for 10 s, 60 °C for 30 s, and 72 °C for 30 s. Melting-curve analysis showed a single peak for each primer pair, confirming primer specificity. *PfActin* was used as the internal reference gene, and its Ct values were controlled between 23 and 28. The relative expression levels of all *PfCAMTA* and *PfCML* genes were calculated using the 2^−ΔΔCT^ method [38] with three biological replicates. Gene-specific primers for all *PfCAMTA* genes are listed in Table S1.

### 4.5. Subcellular Localization of PfCAMTA5

The coding sequence (CDS) without stop codon of the *PfCAMTA5* (KAI3462072.1) from *P. fortunei* was cloned and ligated into the pRI101-GFP vector. The recombinant plasmid pRI101-*PfCAMTA5*-eGFP was introduced into competent *Agrobacterium tumefaciens* cells (GV3101-pSoup). A positive colony was cultured, resuspended in infiltration buffer (10 mM MgCl_2_, 10 mM MES, pH 5.6, and 150 µM acetosyringone) to an OD_600_ of 0.6 for infiltration into *Nicotiana benthamiana* leaves. After 72 h, fluorescence signals were observed using a confocal laser scanning microscope (LSM710, Carl Zeiss AG, Oberkochen, Germany) with an excitation wavelength of 488 nm. For subcellular localization, AtH2B was used as marker. Primers are listed in Table S1.

### 4.6. Transient Overexpression of PfCAMTA5 in Tobacco and Salt Stress Treatment

For functional validation of *PfCAMTA5* in response to salt stress, the recombinant fusion expression vector pRI101-*PfCAMTA5*-GFP and the empty control vector pRI101-GFP were independently introduced into *A. tumefaciens* strain GV3101 (pSoup-p19). The agroinfiltration assay was conducted in *N. benthamiana* at the four-week-old stage (16 h light/8 h dark photoperiod, 150 μmol·m^−2^·s^−1^ light intensity), according to a previously established transient expression protocol with minor modifications [39]. After 72 h, both the *PfCAMTA5*-GFP and pRI101-GFP plants were irrigated once with 200 mmol·L^−1^ NaCl solution (50 mL per plant) to simulate salt stress [40]. Two days after salt treatment, leaf phenotypic symptoms were observed. Subsequently, the infiltrated leaves were collected for the determination of marker gene expression analysis and physiological assays. *NbActin* was used as the internal reference gene and the relative expression level was calculated using the 2^−ΔΔCt^ method. Three independent biological replicates were performed for each treatment.

### 4.7. Determination of Physiological Indices

The enzymatic activities of superoxide dismutase (SOD), peroxidase (POD), and catalase (CAT), as well as malondialdehyde (MDA) content were determined using corresponding assay kits (Solarbio, Beijing, China). The relative electrolyte leakage was measured as reported by Shi et al. [41]. All physiological measurements were performed with three biological replicates.

### 4.8. Homology Modelling and Docking Between PfCAMTA5 and PfCML Proteins

Protein structures were predicted using SWISS-MODEL. For PfCML10 and PfCML28, experimental crystal structures were selected as templates (GMQE = 0.64 and 0.70, respectively). For PfCAMTA5, no suitable experimental template was available; the AlphaFold DB model with the highest GMQE (0.66) was selected. Protein–protein docking was performed on the ZDOCK web server, and the top-ranked pose was analyzed. Binding free energy was calculated with PDBePISA. Non-covalent interactions were characterized using PLIP, with hydrophobic contacts (≤3 Å) and hydrogen bonds (≤3 Å, angle ≥150°) defined by custom cutoffs and other interactions assigned by default criteria. Interface residues were defined as those within 5 Å of the binding partner or involved in the identified interactions.The docking workflow followed the protocol described by Liu et al. [42].

### 4.9. Yeast Two-Hybrid Assays

For the yeast two-hybrid (Y2H) assays, the full-length coding sequences of *PfCAMTA5*, *PfCML10*, and *PfCML28* were cloned into the pGADT7 (AD) and pGBKT7 (BD) vectors to generate the corresponding fusion constructs. Five interaction pairs (pGADT7-PfCAMTA5+pGBKT7, pGADT7+pGBKT7-PfCML10, pGADT7+pGBKT7-PfCML28, pGADT7-PfCAMTA5+pGBKT7-PfCML10, pGADT7-PfCAMTA5+pGBKT7-PfCML28) were co-transformed into the yeast AH109 strain, as previously described [29]. The combinations involving empty vectors (AD + BD-PfCML10, AD + BD-PfCML28, and AD-PfCAMTA5 + BD) were specifically designed as negative controls to exclude autoactivation. The transformed cells were grown on SD/-Trp/-Leu (DDO) dropout plates to select positive co-transformants. Single colonies were picked and cultured in liquid DDO medium until the optical density at 600 nm (OD_600_) reached 1.0. The cultures were then serially diluted (10^−1^, 10^−2^, and 10^−3^) and spotted onto SD/-Trp/-Leu/-His/-Ade selective medium, and incubated at 30 °C for 3–5 days. All experiments were repeated three times independently, yielding comparable results.

### 4.10. Statistical Analysis

Statistical analyses were performed using GraphPad Prism software (v8.0.2). All experiments were conducted with three independent biological replicates. Statistical significance was determined by one-way ANOVA followed by Dunnett’s multiple comparisons test against the EV control for multi-group comparisons, or by two-tailed unpaired *t*-test for pairwise comparisons, as appropriate, with *p* < 0.05 (*), *p* < 0.01 (**) and *p* < 0.001 (***) considered statistically significant.

## 5. Conclusions

In summary, we identified eight *PfCAMTA* genes in the *P. fortunei* genome and classified them into three phylogenetic clades. The eight *PfCAMTA* genes displayed distinct tissue-preferential expression patterns under salt stress, and *PfCAMTA5* exhibited the highest transcript abundance, the most pronounced salt-responsive induction, and the greatest number of predicted post-translational modification sites. Transient overexpression of *PfCAMTA5* in tobacco improved salt tolerance by enhancing antioxidant enzyme activities, photosynthetic capacity, and stomatal behavior, and by up-regulating downstream stress-responsive genes. These findings suggest that *PfCAMTA5* functions as a potential regulator of salt tolerance, providing a candidate gene for molecular breeding of salt-tolerant varieties.

## Figures and Tables

**Table 1:** The physicochemical properties of the paulownia CAMTA proteins.

Gene ID	Gene Name	Genebank ID	Chromosomal Localization	Number of Amino Acids	Molecular Weight (kDa)	Theoretical pI	Grand Average of Hydropathicity	Subcellular Location
Pfo02g014220.1	PfCAMTA1	KAI3445733.1	Chr2	1048	117.28	5.57	−0.448	Nucleus
Pfo04g003860.1	PfCAMTA2	KAI3448258.1	Chr4	1020	114.03	5.63	−0.493	Nucleus
Pfo07g000370.1	PfCAMTA3	KAI3452582.1	Chr7	924	103.65	6.41	−0.425	Cytoplasm/ Nucleus
Pfo11g000790.1	PfCAMTA4	KAI3459232.1	Chr11	899	101.31	8.13	−0.427	Cytoplasm/ Nucleuus
Pfo13g001440.1	PfCAMTA5	KAI3462072.1	Chr13	1114	125.31	5.31	−0.59	Nucleus
Pfo17g007840.1	PfCAMTA6	KAI3467723.1	Chr17	930	104.85	6.85	−0.502	Cytoplasm/ Nucleus
*Pfo19g000270.1*	*PfCAMTA7*	KAI3469225.1	Chr19	962	107.57	6.71	−0.371	Nucleus
*Pfo19g001150.1*	*PfCAMTA8*	KAI3469309.1	Chr19	930	105.43	7.18	−0.479	Nucleus

## Data Availability

No new data were created or analyzed in this study. Data sharing is not applicable to this article.

## Supplementary Materials

The following supporting information can be downloaded at: https://www.mdpi.com/article/10.3390/ijms27219476/s1.

## Abbreviations

The following abbreviations are used in this manuscript:

AP2/EREBP	APETALA2/ethylene-responsive element binding protein
bHLH	Basic Helix-Loop-Helix
CaM	Calmodulin
CDD	Conserved domain database
CDS	Coding sequence
CAT	Catalase
CML	Calmodulin-like protein
CHX	Cation/H^+^ Exchanger
DREB	Dehydration-responsive element-binding protein
GFP	Green fluorescent protein
GELP	GDSL-type esterase/lipase
LTP	Non-specific lipid transfer protein
MYB	Myeloblastosis
MDA	Malondialdehyde
NHX	Na^+^/H^+^ antiporter
NAC	NAM/ATAF1/2/CUC2
POD	Peroxidase
P5CS	△^1^-Pyrroline-5-carboxylate synthetase
RT-qPCR	Quantitative Real-Time PCR
SOD	Superoxide dismutase
SOS	Salt overly sensitive
